# Effects of Space Flight on Inflammasome Activation in the Brain of Mice

**DOI:** 10.3390/cells14060417

**Published:** 2025-03-12

**Authors:** Upal Roy, Roey Hadad, Angel A. Rodriguez, Alen Saju, Deepa Roy, Mario Gil, Robert W. Keane, Ryan T. Scott, Xiao W. Mao, Juan Pablo de Rivero Vaccari

**Affiliations:** 1 Department of Health and Biomedical Science, University of Texas Rio Grande Valley, Brownsville, TX 78539, USA; angel.rodriguez29@utrgv.edu (A.A.R.);; 2 Department of Cellular Physiology and Molecular Biophysics, University of Miami Miller School of Medicine, Miami, FL 33136, USA; 3 Department of Psychological Science and School of Medicine Institute of Neuroscience, University of Texas Rio Grande Valley, Brownsville, TX 78539, USA; 4 Department of Neurological Surgery and the Miami Project to Cure Paralysis, University of Miami Miller School of Medicine, Miami, FL 33136, USA; 5 KBR, Space Biosciences Division, NASA Ames Research Center, Moffett Field, CA 94035, USA; 6 Department of Basic Sciences, Division of Biomedical Engineering Sciences (BMES), Loma Linda University, Loma Linda, CA 92354, USA

**Keywords:** inflammasome, inflammation, RR-1, space biology, International Space Station, innate immunity, inflammation

## Abstract

Space flight exposes astronauts to stressors that alter the immune response, rendering them vulnerable to infections and diseases. In this study, we aimed to determine the levels of inflammasome activation in the brains of mice that were housed in the International Space Station (ISS) for 37 days. C57BL/6 mice were launched to the ISS as part of NASA’s Rodent Research 1 Mission on SpaceX-4 CRS-4 Dragon cargo spacecraft from 21 September 2014 to 25 October 2014. Dissected mouse brains from that mission were analyzed by immunoblotting of inflammasome signaling proteins and Electrochemiluminescence Immunoassay (ECLIA) for inflammatory cytokine levels. Our data indicate decreased inflammasome activation in the brains of mice that were housed in the ISS for 37 days when compared to the brains of mice that were maintained on the ground, and in mice corresponding to the baseline group that were sacrificed at the time of launching of SpaceX-4. Moreover, we did not detect any significant changes in the expression levels of the pro-inflammatory cytokines TNF-α, IL-2, IFN-γ, IL-5, IL-6, IL-12p70 and IL-10 between the ground control and the flight groups. Together, these studies suggest that spaceflight results in a decrease in the levels of innate immune signaling molecules that govern inflammasome signaling in the brain of mice.

## 1. Introduction

Space flight exposes astronauts to a variety of stressors [1,2,3,4,5,6,7,8] ranging from psychological to physical that stem from the environment they experience [9]. These environmental factors include gravity changes, radiation exposure, altered nutrition or altered circadian rhythms, all of which affect the immune response [10,11,12,13,14]. Dysregulation of the immune response has two-fold consequences for astronauts. First, it has the potential to jeopardize the mission while in space, and it causes long-term detrimental consequences [8,15,16], not just while they are in space, but also once they return to Earth [8,17,18,19]. Therefore, it is imperative to gain a better understanding of how the immune response is altered by space flight.

Inflammasomes are supramolecular complexes comprising a sensor molecule such as NOD-like receptor 3 (NLRP3), the adaptor protein apoptosis-associated speck-like protein containing a caspase recruitment domain (ASC), and the cysteine aspartase caspase-1 [20]. The inflammasome plays a significant role in the regulation of the inflammatory response [21,22,23,24,25] by modulating the innate immune response through the activation of caspase-1, the processing of the pro-inflammatory cytokines interleukin (IL)-1β and IL-18, as well as the programmed cell death mechanism of pyroptosis [26]. The inflammasome has been shown to contribute to a variety of diseases including neurodegenerative diseases such as multiple sclerosis [27,28,29,30,31], Parkinson’s disease [32,33], Alzheimer’s disease [34,35], inflammaging [36,37] and metabolic syndrome [38,39]. Several of these diseases, with an inflammasome etiology, have also been suggested to be relevant in the context of space flight such as the development of dementia [40], altered aging and inflammation [41] or diabetes [42]. Hence, it is important to understand the effects of space flight on inflammasome signaling to develop therapies against specific targets of the immune response to protect and improve the performance of astronauts not only during a mission, but also their health on Earth [43,44].

It has been shown that space radiation affects hippocampus-dependent cognitive tasks [45], spatial navigation and episodic memory formation [46,47], which is very important for the overall health of astronauts [43,48,49,50,51,52,53,54,55,56,57]. In addition, it has been suggested that space radiation affects the cortex and causes altered cognitive flexibility and synaptic plasticity, which contribute to cognitive deficits [58]. A recent study used the anti-oxidant LGM2605 to inhibit the NLRP3 inflammasome in an in vitro model of lung vascular networks that were exposed to γ-rays and mixed-field radiation sources to mimic some of the space flight conditions in vitro [59]. The study found that 3–4 keV/µm and 8–10 keV/µm linear energy transfer (LET) proton radiation elevated the levels of NLRP3, and that this increase can be targeted with anti-oxidant approaches [59]. In this study, we aimed to characterize the effect of space flight on the expression of inflammasome proteins and inflammatory cytokines in mice that were part of NASA’s Rodent Research 1 Mission (RR-1) on SpaceX-4 CRS-4 Dragon cargo spacecraft that spent 37 to 38 days at the International Space Station (ISS).

## 2. Materials and Methods

Animal procedures were approved by IACUC numbers NASA: NAS-13-002-Y1 and CASIS: CAS-13-001-Y1 for flight at the NASA Ames Research Center and the Kennedy Space Center in accordance with relevant guidelines and regulations.

### 2.1. Flight Animals

**Figure 1:**
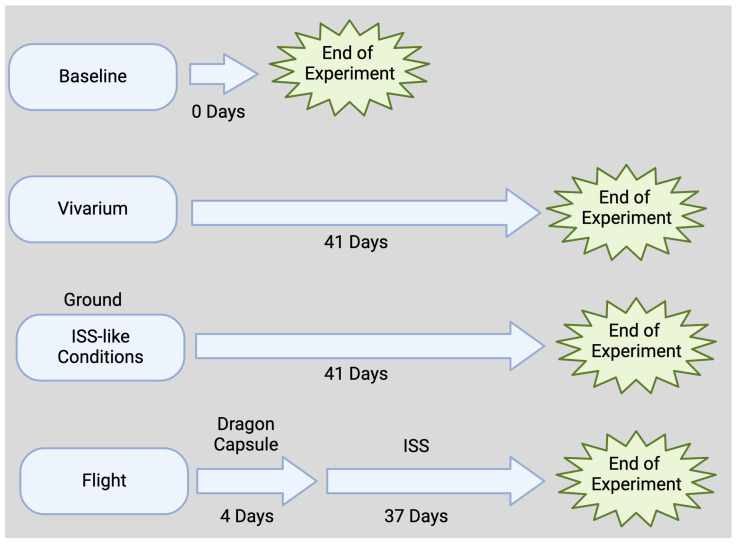
Study timeline.

This animal study was approved by the NASA Ames Research Center and The Kennedy Space Center Institutional Animal Care and Use Committees. Brain tissue samples were obtained by the authors through an award from the NASA Biological Institutional Scientific Collection. C57BL/6 female mice (16-week-old, Jackson Labs) were flown to the ISS as part of RR-1 on SpaceX-4 CRS-4 Dragon cargo spacecraft [60]. During the flight, animals were kept at an ambient temperature of 26 to 28 °C in a 12 h light and dark cycle. Flight animals received water and a food bar diet specifically designed by NASA ad libitum. RR-1 mission animals were on board the Dragon capsule for 4 days (transit time) and then spent 37 days in the ISS (Figure 1).

RR-1 (https://osdr.nasa.gov/bio/repo/data/payloads/RR-1, accessed on 9 March 2025) lasted 37 days, and according to the NASA Open Science Data Repository′s (OSDR) Environmental Data App, between 21 September and 23 September, these mice experienced temperature changes ranging from 18.3 °C to 26.69 °C. On 24 September, the temperature stabilized between 21 and 23 °C. Importantly, mice in the ISS (flight group) and in the ground control group experienced the same temperature conditions. Overall, the lowest temperature in the ISS during RR-1 was 18.50 °C and the highest temperature was 26.80 °C, with a mean temperature of 22.76 °C, whereas in the ground control group, the lowest temperature was 18.30 °C and the maximum was 27.10 °C (mean temperature 22.76 °C). Moreover, telemetry data indicated that mice in the flight group experienced initial higher CO_2_ levels at around 2621 ppm on 20 September, whereas mice in the ground group experienced much lower CO_2_ levels at approximately 476 ppm. By 27 September, both groups of mice experienced similar CO_2_ conditions through the rest of the flight, approximately between 1868 ppm and 4690 ppm. The minimum CO_2_ level in the ISS group was 1862 ppm and the maximum was 4667 ppm (mean 3197.27, median 3192 ppm), whereas in the ground group, the minimum was 438 ppm, the maximum was 4690 ppm and the mean was 2853.74. The median was 3188 ppm. Moreover, by October 25th, mice in the ISS experienced an accumulated radiation dose of 7.17 mGy/day. In the ISS group, the minimum galactic cosmic radiation (GCR) dose was 0.13 mGy/day, and the maximum was 0.14 mGy/day (mean 0.13 mGy/day). Furthermore, the radiation experienced as a result of the South Atlantic Anomaly (SAA) ranged between 0.06 and 0.10 mGy/day (mean 0.07 mGy/day). Together, these accounted for a minimum total dose of 0.19 mGy/day, a maximum of 0.23 mGy/day and an average of 0.20 mGy/day.

### 2.2. Ground Control Animals

Animals were kept in similar conditions to flight animals (ambient temperature of 26 to 28 °C in a 12 h light and dark cycle). Telemetry data (with a 4-day delay) from the flight group were used to keep the ground control animals under similar conditions (housed in RR-1 flight hardware within an environmental simulator under the same conditions as the ISS). Water and food conditions for this group were the same as for the flight animal group as described.

### 2.3. Vivarium and Baseline Animals

Animals in the vivarium group were animals that were housed under standard vivarium conditions and remained alive for the duration of study. Animals in the baseline group consisted of mice that were euthanized at the time when the animals in the flight group were launched.

### 2.4. Brain Dissection

Within 4 h of splashdown, flight and ground control animals were sacrificed and brains were removed and stored at −80 °C until further analysis. For the study focusing on the forebrain, microdissections of the entire hippocampus and primary motor cortex were performed following well-established rodent brain microdissection protocols [61,62,63], and a brain atlas served as a reference to ensure accuracy of brain region identification [64]. Following brain dissection, samples were stored at −80 °C until further analysis. Tissues used in this study consisted of brain cortex, hippocampus and rest of the tissue from the brain areas are referred as “other than cortex and hippocampus”.

### 2.5. Immunoblotting

Analysis of inflammasome signaling proteins in brain protein lysates by immunoblotting was carried out as described in [65]. Accordingly, brain lysates (25 μg) were run and resolved in 4–20% Criterion TGX Stain-Free precast gels (Bio-Rad, Hercules, CA, USA) prior to transfer to PVDF membranes (Bio-Rad). Membranes were then incubated with primary antibodies against caspase-1 (Novus Biologicals Centennial, CO, USA), ASC (Santa Cruz Biotechnology, Inc., Dallas, TX, USA), and IL-1β (Cell Signaling, Technology, Inc., Danvers, MA, USA) at a dilution of 1:1000 for 1 h followed by HRP-linked secondary antibodies (1:1000) for 45 min. β-actin (1:5000, Sigma Aldrich, St. Louis, MO, USA) was used as a standard and protein loading control. Membranes were imaged with the ChemiDoc Touch Imaging System (Bio-Rad) following enhanced chemiluminescence. Densitometric analysis for relative protein quantification was carried out with UN-SCAN-IT gel 6.3 Software (Silk Scientific Corporation, Vineyard, UT, USA).

### 2.6. Electrochemiluminescence Immunoassay (ECLIA)

Using protein lysates, a V-PLEX ECLIA kit (MSD) was used to measure the inflammatory profile in the different brain sections as described in [66,67] using the MESO-QuickPlex SQ-120MM (MSD, Rockville, MD, USA). Briefly, to measure the protein levels of TNF-α, IL-2, IFN-γ, IL-5, IL-6, IL-12p70 and IL-10, after washing steps, the plate was incubated overnight with 50 µL of samples. The following day, after washing steps, samples were incubated in detection antibodies for 2 h followed by 3 more washing steps and the addition of the 2X Read Buffer (MSD, Rockville, MD, USA). After obtaining the quantity of protein in pg/mL for each analyte using the MESO-QuickPlex SQ-120MM, samples were then normalized to total protein concentration per sample.

### 2.7. Statistical Analyses

Statistical analyses were carried out with Prism 10.0 software (GraphPad Software, San Diego, CA, USA). Descriptive statistics were first calculated. Data were tested for normality with the Shapiro–Wilk Test. Comparisons between the flight and ground groups were carried out by a one-tail *t*-test when data met normality and by the Mann–Whittney test when data were not normally distributed.

## 3. Results

### 3.1. Levels of Inflammasome Signaling Molecules Are Decreased in the Brain of Mice That Experienced Spaceflight

**Figure 2:**
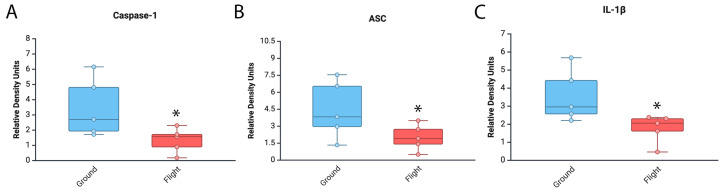
Inflammasome signaling proteins in the brain of mice in the ISS. Immunoblot of inflammasome proteins for the expression of (**A**) caspase-1, (**B**) ASC and (**C**) IL-1β in an area of the brain of mice other than cortex and hippocampus after 37 days of spaceflight. Data were normalized to β-actin as a protein loading control. Data presented as box-plots with dots as all data points. * *p* < 0.05. N = 5 per group.

It has been previously shown that space travel affects the immune response [11]. To test the effects of spaceflight on inflammasome activation in the brain, we carried out an immunoblot analysis of brain areas other than cortex and hippocampus from mice that were in the ISS for approximately 1 month (Figure 1). Our findings indicate that caspase-1 (95% CI = Ground [1.06–5.9], Flight [0.3–2.3]) (Figure 2A), ASC (Ground [1.3–7.6], Flight [0.6–3.5]) (Figure 2B) and IL-1β (Ground [1.8–5.4], Flight [0.8–2.7]) (Figure 2C) were significantly decreased in the mice that were in the ISS when compared to the ground control group. In addition, the expression levels for all the inflammasome proteins analyzed in these studies in the vivarium group and those in the baseline group were similar to the spaceflight group (Figure S1). Together, these findings indicate that the expression levels of inflammasome signaling proteins in the brain of mice are lower in the mice that were in the ISS (flight) than the mice in the ground control group.

### 3.2. Inflammasome Proteins Are Decreased in the Cortex and Hippocampus of Mice After Spaceflight

**Figure 3:**
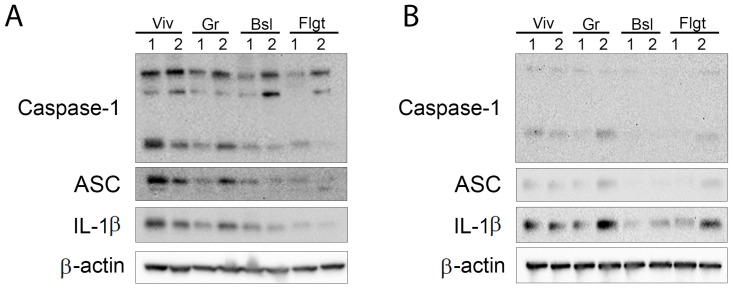
Inflammasome signaling proteins in the (**A**) cortex and (**B**) hippocampus of mice in the ISS. Immunoblot of inflammasome signaling proteins caspase-1, ASC and IL-1β in the cortex of mice after 37 days of spaceflight. Viv = Vivarium, Gr = Ground, Bsl = Baseline and Flgt = Flight. Data were normalized to β-actin as a protein loading control. N = 2 per group.

We then aimed to determine the levels of inflammasome signaling proteins present in the cortex (Figure 3A) and hippocampus (Figure 3B). Accordingly, immunoblot analysis of cortical and hippocampal protein lysates suggested that similar to our previously described findings (Figure 2), there was a trend towards decreased inflammasome activation in the cortex (Figure 3A) and the hippocampus (Figure 3B) of mice after spaceflight when compared to the ground control group. In addition, the brains of mice in the baseline group also had significantly lower levels of inflammasome activation in the cortex and hippocampus, which were similar to the protein expression levels in the flight group. Moreover, animals in the vivarium group presented much higher inflammasome protein levels than under baseline and spaceflight conditions. These findings suggest that in the cortex and hippocampus, inflammasome activation is lower after spaceflight.

### 3.3. Levels of Tnf-α and Il12p70 Are Decreased in the Brain of Mice After Spaceflight

**Figure 4:**
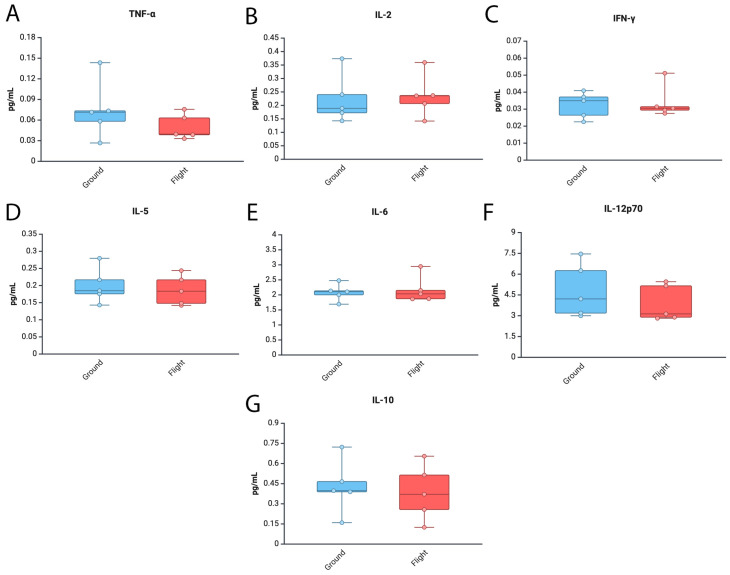
Inflammatory cytokine profiles in the brains of mice in the ISS. Protein levels of inflammatory cytokines (**A**) TNF-α, (**B**) IL-2, (**C**) IFN-γ (**D**) IL-5, (**E**) IL-6, (**F**) IL-12p70 and (**G**) IL-10 in an area of the brain of mice other than cortex and hippocampus after 37 days of spaceflight and measured by ECLIA. Data were normalized to total protein. Data presented as box-plots with dots as all data points. N = 5 per group.

Gene expression changes associated with inflammation have been described in the brains of mice who experienced spaceflight conditions and remained in the ISS for over 30 days [61]. To determine the protein levels of inflammatory cytokines in the brain of mice in the ground control and spaceflight conditions, we carried out an ECLIA to measure the protein levels of TNF-α (Figure 4A), IL-2 (Figure 4B), IFN-γ (Figure 4C), IL-5 (Figure 4D), IL-6 (Figure 4E), IL-12p70 (Figure 4F) and IL-10 (Figure 4G) in brain areas other than the cortex and hippocampus. Accordingly, we found similar levels of these cytokines between the ground and flight groups (Figure 4). Moreover, there was a trend towards lower levels of TNF-α and IL-12p70 in the baseline group when compared to the other groups (Figure S2). Together, these trends suggest that these inflammatory cytokines were not altered in regions outside the cortex and hippocampus as a result of mission RR1.

### 3.4. Inflammatory Cytokine Levels in the Cortex and Hippocampus of Mice After Spaceflight

To preliminarily determine the inflammatory profile in the cortex (Figure S3) and hippocampus (Figure S4) of mice, we used ECLIA to measure the levels of TNF-α, IL-10, IFN-γ, IL-5, IL-6 and IL-12p70 in protein lysates. However, due to the low sample size (N = 2), we were unable to carry out any statistical analyses in these tissues for the expression of these cytokines, yet the preliminary findings suggest that in the brain, the inflammatory response mediated by different cytokines is different in the cortex (Figure S3) than in the hippocampus (Figure S4) as a result of space flight.

## 4. Discussion

Previous studies have shown that the adaptive immune response is compromised as a result of space flight missions, resulting in the reactivation of viruses [68]. In addition, a previous study has shown that following 13 days of spaceflight on board of space shuttle mission STS-118, mice experienced a decrease in liver, spleen and thymus mass compared to the ground control group, which was consistent with the decreased number of lymphocytes, monocyte/macrophage and granulocytes [69]. However, how space flight affects the inflammatory response mediated by the inflammasome in the brain of mice has yet to be fully understood. In this study, we determined the levels of the key inflammasome signaling proteins caspase-1, ASC and IL-1β in the brain of mice after a stay of 37 days on the ISS. In addition, we measured the levels of cytokines that contribute significantly to the inflammatory response in the brain such as TNF-α, IL-10, IFN-γ, IL-5, IL-6 and IL-12p70 [70,71,72]. We used immunoblotting and ECLIA approaches to determine the inflammatory protein profile in the brains of mice that were in the ISS as part of the RR-1 mission that was launched on SpaceX-4 CRS-4 on 21 September 2014 and returned to earth on October 25th of the same year. Consistent with results from the STS-118 study, here we demonstrate that conditions experienced during a mission to low Earth orbit result in a significant decrease in levels of inflammasome proteins in the brain.

Previous studies from RR-1 have shown that these mice experienced increased expression of genes associated with non-shivering thermogenesis in interscapular brown adipose tissue as well as increased expression of genes associated with glucose metabolism and adipogenesis in gonadal white adipose tissue [73], consistent with other studies that showed altered liver [74] and muscle metabolism in these mice, which suggested that these mice experienced muscle atrophy [75] and that dietary changes might have been needed as a countermeasure [76].

During the RR-1 mission, mice in the flight group presented higher levels of CO_2_ than the ground control group (2621 ppm vs. 476 ppm). However, approximately a week later, mice in both groups experienced similar CO_2_ conditions. Increased CO_2_ levels in neutrophils for a couple of hours have been shown to increase IL-1β as well as the expression of NLRP3 and caspase-1 without affecting the levels of ASC [77]. Thus, it is possible that the changes seen between the ground control group and the flight group are due to the fluctuations in the CO_2_ levels. Furthermore, another variable that needs to be taken into account is that there was a difference between the vivarium group and the ground control group, bringing up the possibility of an effect due to the habitat. Notably, the CO_2_ levels in the vivarium group were the same as ambient air (~419 ppm), which were much lower than the ground group (median: 3188 ppm). In addition, some differences have been described between the vivarium and the animal enclosure module in which water and food consumption were higher in the animal enclosure module without a significant effect on overall body mass but some differences in the weight of certain organs were reported; however, that study did not report the weight of the brain [78]. Alternatively, taken together, it is possible that the effects seen in the flight group are in part due to the effects of the CO_2_ levels in the brain and the stress associated with space flight. Therefore, part of the elevated levels seen in the ground control group compared to the vivarium group may be due in part to the habitat environment and the CO_2_ levels. Importantly, the CO_2_ levels between the ground and the flight groups eventually stabilized after 7 days (ISS median: 3192 ppm; ground median: 3188 ppm).

During the RR-1, mice in the flight group experienced an average of 0.20 mGy/day. Previous studies have shown that whole body exposure to higher doses from a ^137^CsCl radiation source (0.5 to 4 Gy) resulted in a dose-dependent increase in inflammasome activation as determined by the cleavage of caspase-1 at 1 day after exposure, an effect that was not present at 1 and 4 h; moreover, caspase-1 cleavage was significantly decreased by day 14 in immune cells [79]. Following this radiation protocol, inflammasome activation was mainly identified in macrophages, dendritic cells, and natural killer cells [79]. In addition, UV radiation has been shown to also activate inflammasomes [80]. Furthermore, it has been shown that exposure of mice to silicon (0.5 Gy) or iron (0.15 Gy) ions results in increased cytokine production that is associated with infiltration of immune cells [81,82]. A recent study in a model of the lung vascular network exposed to low levels of radiation ranging from 0.25 to 1 Gy [83] showed increased expression of the inflammasome sensor NLRP3, which was decreased by treatment with LGM2605, a scavenger of radiation-induced reactive oxygen species (ROS) and active chlorine species (ACS), which is capable of reducing ionizing species due to radiation [59]. Similarly, in a study using a clinostat to model microgravity (10^−3^ gravitational force) in human umbilical vein endothelial cells, the authors showed that microgravity also increased the expression of NLRP3 consistent with increased expression of IL-1β and increased stress in the endoplasmic reticulum [84]. Moreover, a recent study showed that 8 Gy of radiation can inhibit activation of the NLRP3 inflammasome in macrophages exposed to ATP and LPS by a mechanism that involves inhibiting TWIK2 expression [85]. However, whether the 7.17 mGy/day of accumulated radiation is also capable of causing lower levels of inflammasome protein expression experienced by mice in the flight group is yet to be tested.

In this study, we have shown that in the brain of mice who were in the ISS, there were lower levels of the inflammasome signaling proteins caspase-1, ASC and IL-1β when compared to the ground control group. Interestingly, the levels of these proteins were similar to those levels present in the brains of the animals corresponding to the baseline group, which were sacrificed at the time of launch, suggesting that the inflammasome proteins continued to increase over time in the ground control group; however, this increase in inflammasome protein expression was arrested or stalled in the ISS group. We have previously shown that inflammasome activation in the brain increases as part of the inflammaging response [36,37,67]. In contrast to those inflammaging studies, the brain data from the mice in RR1 presented lower levels of these inflammasome proteins similar to those levels that were present in mice that were sacrificed at the time of launch. These data would suggest that the innate immune response mediated by the inflammasome is not weakened by space flight, but that it does not develop as it normally does on Earth. Consistent with these findings on decreased inflammation in the Central Nervous System (CNS), animals in RR-1 have also been shown to present younger epigenetic age in the retina than the ground control group [86]. Similarly, in the NASA Twin Study, the spaceflight twin presented a longer telomere length than his twin brother who remained on Earth, suggesting that in space, the aging process is slowed down [41]. Together, these findings support our hypothesis that spaceflight decreases brain inflammaging mediated by the inflammasome.

It is possible that the decrease in the levels of inflammasome signaling proteins after spaceflight may be the result of a protective attempt to dampen the innate immune response in the brain following the stress experienced in space in the ISS. Stalled replication forks have been previously shown to limit the innate immune response mediated by cGAS–STING following replication stress and in order to maintain genome stability [87]. Thus, it is possible that the stress of exposure associated with radiation and microgravity [88,89,90,91,92,93] in the ISS mice resulted in stalled replication forks responsible for regulating inflammasome expression in the brain, which ultimately is responsible for the levels of inflammasome activation in the brain similar to those seen in mice that were sacrificed at the time of launch (baseline group).

Furthermore, the inflammatory cytokines tested presented a similar pattern of expression between the baseline and flight groups. Moreover, our findings on the cortex and hippocampus suggest a differential expression of different cytokines in different parts of the brain, even with a very limited sample size. As anticipated, these findings suggest diverse roles in the inflammatory response mounted by different cytokines in the brain.

In general, we would have anticipated that the inflammasome would be increased in the ISS group when compared to the baseline group based on previous studies looking at the peripheral immune response as a result of space flight. However, in this study, we have measured the inflammatory response in the CNS, suggesting that the immune response mounted in the brain as a result of space flight differs between the CNS and the periphery, an effect that is perhaps due to the relatively immune-privileged status of the brain owing to the characteristics of the blood–brain barrier. Moreover, similar observations have been previously reported in which spaceflight conditions resulted in immune dysfunctions and dysregulated inflammation [94,95]. Future studies are needed to confirm these results in other missions. However, consistent with these results, a previous study in the brain of mice that were in the ISS for 35 days and were flown on board SpaceX-12 as part of RR9 showed downregulation of immune genes [61]. Furthermore, in this study, only female mice were used. Thus, future studies are needed to better understand the sex differences associated with space flight in the inflammatory response in the brain, considering that previous studies have shown that brain cells respond differently in males vs. females to space-related radiation conditions [96,97,98,99]. Another limitation of this study is the low sample size characteristic of this type of space biology studies. In addition, although the trends are similar across different brain areas, it is not clear what areas of the brain are included in those that did not include the cortex and hippocampus. Moreover, since we have only analyzed samples from RR-1, we were not able to compare the results of RR-1 to other missions. Thus, we do not know if the results of this study could be generalized to space flight in general or if these findings are particular to events/conditions that took place during the RR-1 mission. Future, studies should aim at analyzing mouse brains from other missions with larger sample sizes, as well as studies with more accurately described dissected brain areas, and with considerations for sex differences. Furthermore, it would be important to compare these findings in the rodent brain to blood-based biomarkers obtained from astronauts in missions of different durations.

In conclusion, this study offers for the first time information on the effects of spaceflight on the innate immune response mediated by the inflammasome in the mouse brain after inhabiting the ISS for approximately one month. Thus, it is important to expand on these findings in the future to develop effective countermeasures against the detrimental effects associated with space flight.

## Data Availability

Data produced for this publication were deposited in the NASA Open Science Data Repository (OSDR) under accession numbers OSD-738 (https://doi.org/10.26030/y2bh-8d88), OSD-751 (https://doi.org/10.26030/wa2p-cn71), and OSD-752 (https://doi.org/10.26030/3zzw-1v11). Data used in this paper were downloaded from NASA′s Environmental Data Application (EDA) at the Open Science Data Repository (https://visualization.osdr.nasa.gov/eda/, accessed on 9 March 2025). Data were derived from the Rodent Research-1 (RR1) payload.

## Supplementary Materials

The following supporting information can be downloaded at https://www.mdpi.com/article/10.3390/cells14060417/s1: Figure S1: Inflammasome proteins in the brain. Figure S2: Inflammatory cytokines in the brain. Figure S3: Inflammatory cytokine profile in the cortex of mice in the ISS; Figure S4: Inflammatory cytokine profile in the hippocampus of mice in the ISS.
