# Supplementary material for: Effects of Space Flight on Inflammasome Activation in the Brain of Mice
Source: Cells. 2025 Mar 12;14(6):417. doi: 10.3390/cells14060417 (PMC11941215; doi:10.3390/cells14060417)
Supplement: Supplementary file 1 [file cells-14-00417-s001.zip › cells-3424175-supplementary.pdf]

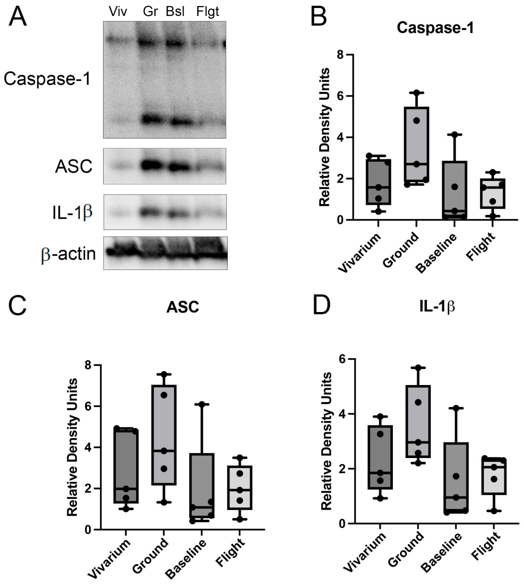

Figure S1. Inflammasome signaling proteins in the brain of mice in the ISS. A) Representative immunoblot of inflammasome proteins for the expression of **B)** caspase-1, **C)** ASC and **D)** IL-1β in an area of the brain of mice other than cortex and hippocampus. Viv= Vivarium, Gr= Ground, Bsl= Baseline, Flgt= Flight. Data were normalized to β-actin as a protein loading control. Data presented as box-plots with dots as all data points. N= 5 per group.

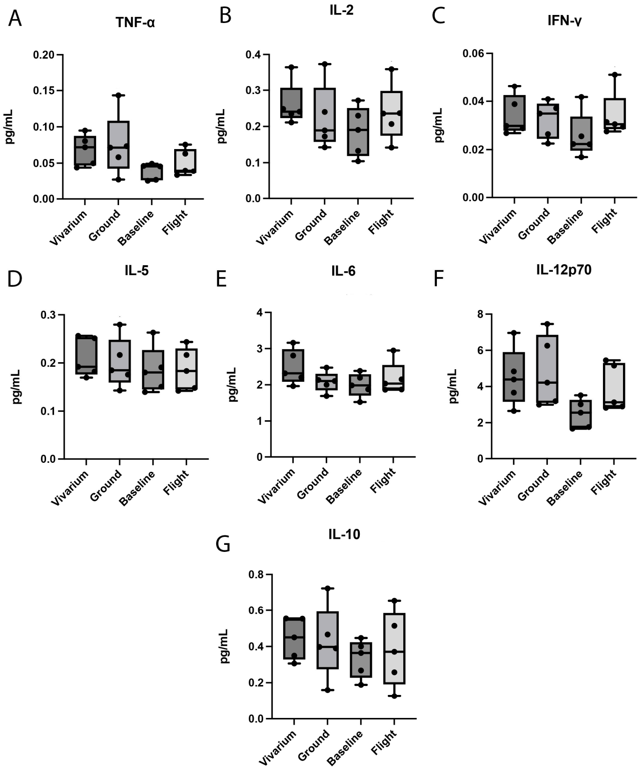

Figure S2. Inflammatory cytokine profile in the brain of mice in the ISS. Protein levels of inflammatory cytokines **A)** TNF-α, **B)** IL-2, **C)** IFN-γ **D)** IL-5, **E)** IL-6, **F)** IL-12p70 and **G)** IL-10 in an area of the brain of mice other than cortex and hippocampus and measured by ECLIA. Data were normalized to total protein. Data presented as box-plots with dots as all data points. N= 5 per group.

CORTEX

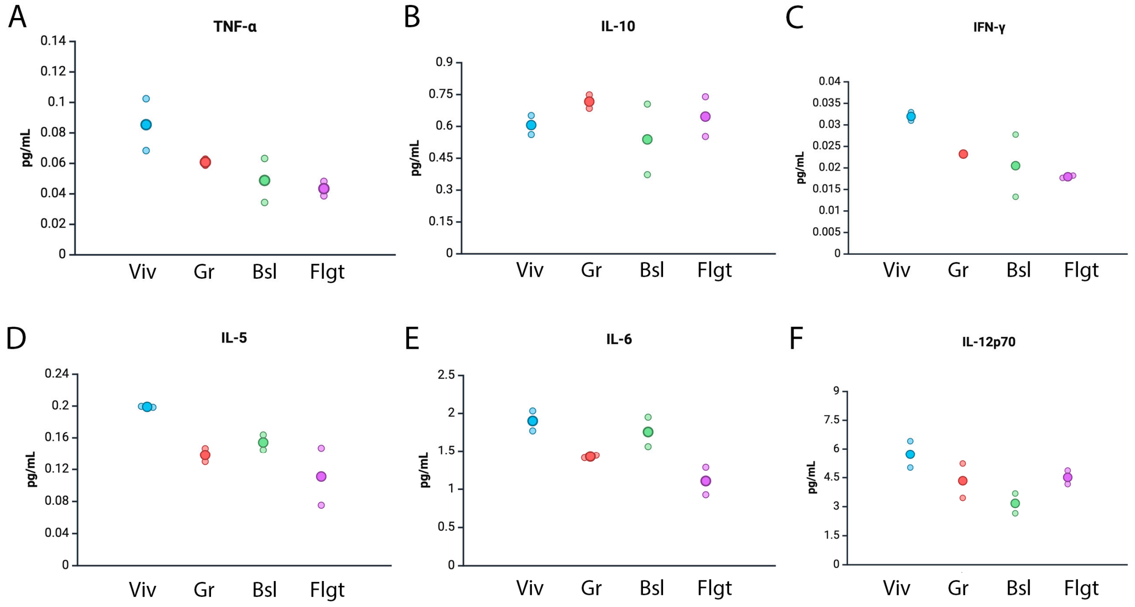

Figure S3. Inflammatory cytokine profile in the cortex of mice in the ISS. Protein levels of inflammatory cytokines **A)** TNF-α, **B)** IL-10 **C)** IFN-γ **D)** IL-5, **E)** IL-6 and **F)** IL-12p70 in the cortex of mice by ECLIA. Data were normalized to total protein. Data presented as median with dots as individual values. N= 2 per group.

HIPPOCAMPUS

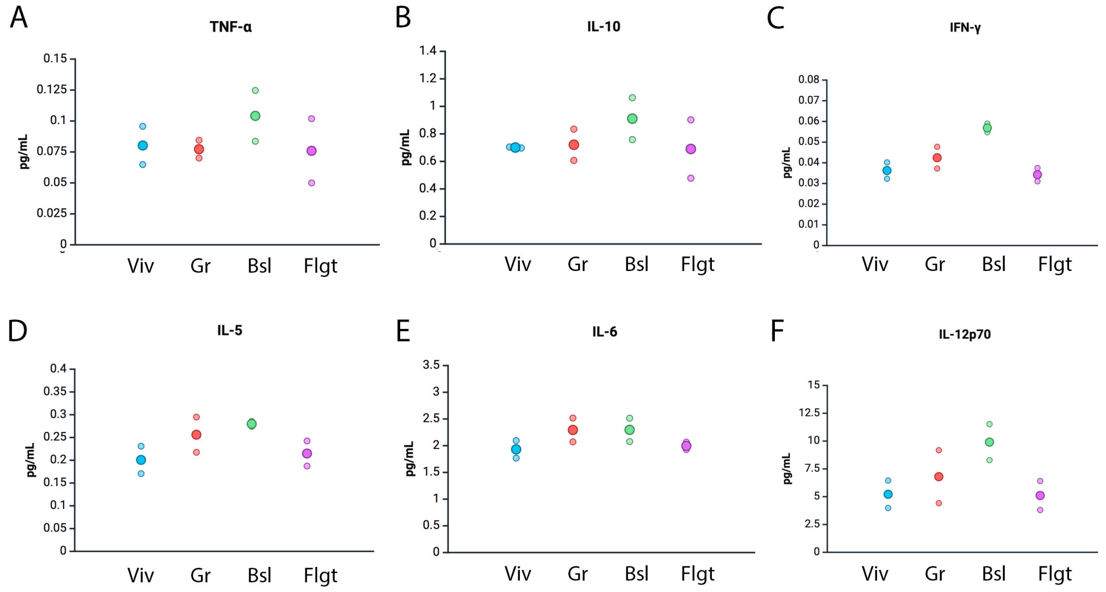

Figure S4. Inflammatory cytokine profile in the hippocampus of mice in the ISS. Protein levels of inflammatory cytokines **A)** TNF-α, **B)** IL-10 **C)** IFN-γ **D)** IL-5, **E)** IL-6 and **F)** IL-12p70 in the hippocampus of mice by ECLIA. Data were normalized to total protein. Data presented as median with dots as individual values. N= 2 per group.
